# Prevalence of gastrointestinal helminths and parasites in smallholder pigs reared in the central Free State Province

**DOI:** 10.4102/ojvr.v86i1.1687

**Published:** 2019-04-11

**Authors:** Ifeoma C. Nwafor, Hester Roberts, Pieter Fourie

**Affiliations:** 1 Department of Agriculture, Faculty of Health and Environmental Sciences, Central University of Technology, Free State, South Africa; 2 Department of Life Sciences, Faculty of Health and Environmental Sciences, Central University of Technology, Free State, South Africa

## Abstract

Pigs are kept by farmers as a source of livelihood and food. Unfortunately, helminthiasis and other internal parasites are major setbacks to profitable pig production in Africa. There is a lack of information on the prevalence and intensity of gastrointestinal helminths and parasites plaguing resource-poor pig farmers in the Free State. Knowledge of these endemic parasites can be used as baseline data to help design future intervention plans. The aim of this study was to identify and quantify the types of gastrointestinal helminths and parasites prevalent in smallholder pigs reared in the central Free State Province. Faecal samples were randomly collected from 77 pigs and parasitologically analysed. Quantification was done using the McMaster counting technique. Farming system, age, gender and health status were the risk factors considered. The study was conducted between January and March 2016. Overall, results showed that 61 samples (79.2%) tested positive for one or more gastrointestinal parasites, which were observed as single or mixed infections. Amongst the positive samples, 44.5% were infected with *Ascaris suum*, 50.6% with *Trichuris suis*, 26.0% and 72.7% were infected with *Oesophagostomum dentatum* and *coccidia*, respectively. There were significant differences (*p* < 0.05) between the rate of infection in the intensive and semi-intensive systems and between the dewormed and non-dewormed pigs. Piglets and female pigs recorded a higher prevalence in their categories. Pigs excreted mostly low (eggs per gram [EPG] ≤ 100) to moderate (EPG > 100 < 500) levels of helminth eggs. It is concluded that different species of gastrointestinal parasites are present in most pigs reared by smallholder farmers in this study area.

**Keywords:** gastrointestinal helminths and parasites; smallholder pig farmers; pigs; prevalence; Central Free State Province.

## Introduction

Globally, pork is the most consumed of all meat products, and South Africa accounts for the highest pig population in southern Africa (Davids et al. 2013). According to Krecek et al. (2004), 25% of pigs produced in South Africa, especially in resource-poor rural areas, are free ranging. In the central Free State Province, pigs are normally reared intensively, semi-intensively or extensively. The intensive system of production is typically practised by established commercial farmers, who account for the bulk of pig production in the province, while the semi-intensive and extensive systems are commonly practised by medium-scale, smallholder or emerging pig farmers (Van Niekerk, Groenewald & Zwane 2014). Pigs are mainly kept as a source of livelihood to generate income and supply protein.

Increasing productivity and sustainability in all farming systems is basically the challenge of all agricultural developments, especially in rural settings. Poor feed conversion ratio, poor reproductive performance, land pollution, parasitic contamination, high morbidity and mortality rates, poor carcass quality and the increased rate of zoonotic infections in humans (e.g. Cysticercosis) are some of the prevalent constraints associated with outdoor pig production systems (Githigia et al. 2005; Kagira 2010; Krecek et al. 2004). Helminthiasis and intestinal parasitism have been reported as major setbacks to profitable pig production in Africa. Various prevalence levels of *Ascaris suum, Strongyloides* spp., *Metastrongylus* spp., *Trichuris suis, Taenia solium, Hyostrongylus rubidus, Fasciolopsis buski*, coccidia spp. and so on have been reported in pigs by different authors (Dey et al. 2014; Marufu et al. 2008; Nsoso et al. 2000; Obonyo et al. 2012; Sowemimo et al. 2012). The infection levels on farms where pigs are bred intensively are usually lower and manifest with less intensity when compared to the traditional systems where poor hygiene, poor nutrition and inadequate anthelmintic interventions favour the proliferation of intestinal helminths (Dey et al. 2014; Nganga, Karanja & Mutune 2008; Tamboura et al. 2006).

Farmers experience both direct and indirect economic losses because of helminthiasis, which also culminates in reduced welfare issues in pigs. Internal parasitism in pigs can result in loss of appetite, poor growth rate, poor feed conversion ratio, organ and carcass condemnation, high cost of treatment and may synergistically increase the effect of other pathogens or even death in severe cases (Kagira et al. 2012). Parameters such as sex, age, management system, season, geographical location and level of farmer awareness have been reported to be risk factors that may influence the various levels of parasitism in live or slaughtered pigs. In addition, some of these parasites are zoonotic in nature and have the potential to cause significant health threats in infected humans (Zoli et al. 2003). Epidemiologic studies by Mafojane et al. (2003), Phiri et al. (2003), Krecek et al. (2004) and Krecek et al. (2012) demonstrated that the highest levels of human neurocysticercosis, which is a zoonotic infection caused by porcine *T. solium*, occurred in the smallholder farming areas of the Eastern Cape Province of South Africa.

To our knowledge, there is a dearth of information on the prevalence, intensity and risk factors of pig helminths and parasites in different types of pig management systems in the central Free State Province. Therefore, the aim of this study was to identify and quantify the types of gastrointestinal helminths and parasites prevalent in smallholder pigs raised in selected districts in the central Free State Province of South Africa. Extensive scholarly knowledge of these endemic parasites in this province can be used as baseline data to help design future effective and sustainable intervention plans.

## Materials and methods

### Description of study area

**FIGURE 1:**
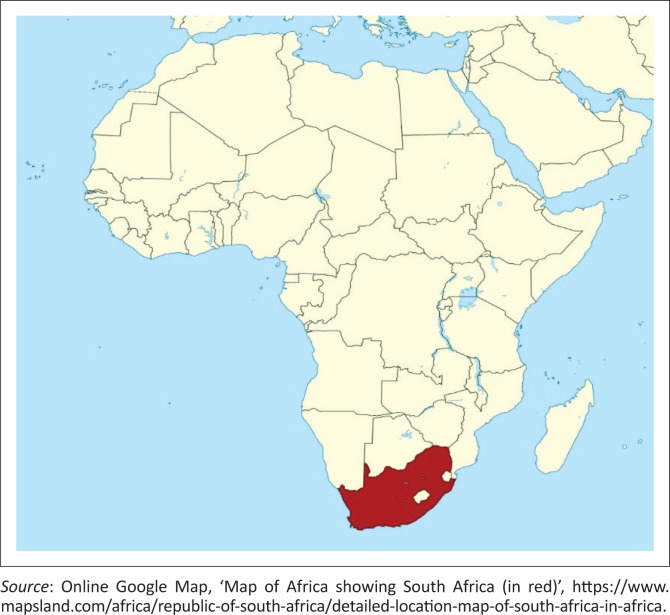
Map of Africa showing South Africa (in red).

**FIGURE 2:**
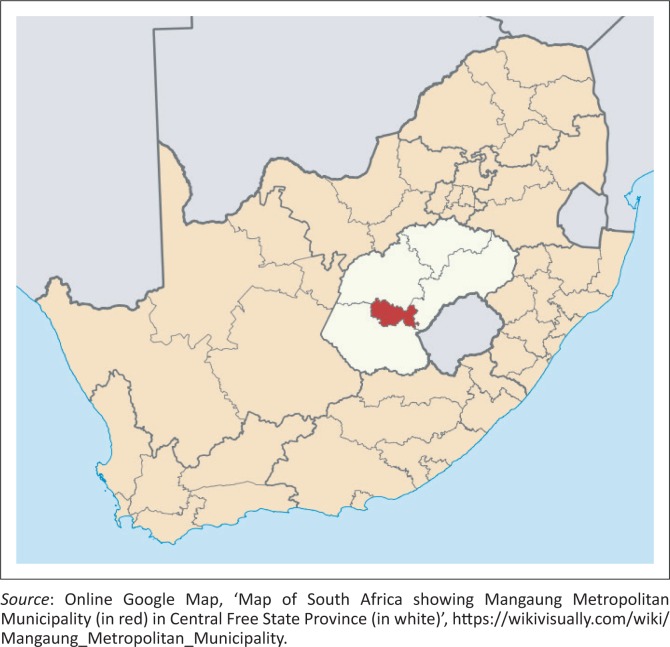
Map of South Africa showing Mangaung Metropolitan Municipality (in red) in the central Free State Province (in white).

**FIGURE 3:**
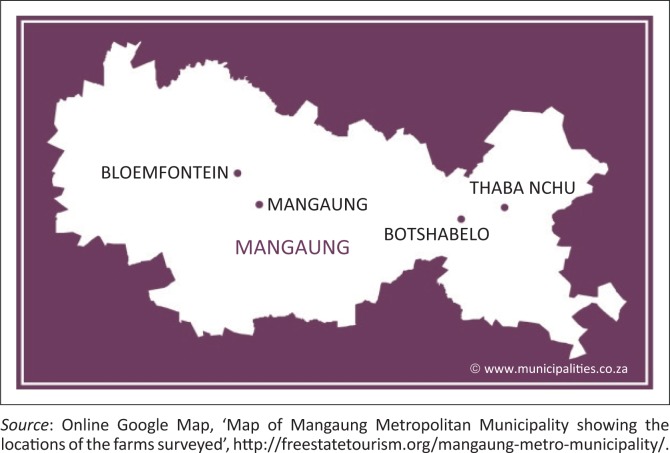
Map of Mangaung Metropolitan Municipality showing the locations of the farms surveyed.

This field study was conducted in four farming communities (Bloemfontein, Botshabelo, Thaba Nchu and Mangaung) in the Mangaung Metropolitan Municipality in the central Free State Province of South Africa. This municipality is located within the coordinates 29°S and 26°E and comprises a geographical area of 4284 km^2^. The area experiences a semi-arid climate with temperatures ranging between 19 °C and 32 °C in the summer months and from –3 °C to 14 °C in the winter months. Annual precipitation varies between 500 mm and 600 mm. The altitude is 1395 metres (45 776 feet [ft]) above sea level (Maphalla & Salman 2002). The geographical locations of the survey farms from a broader to a narrower viewpoint are shown in Figures 1, 2 and 3.

### Sample collection

About four grams of faeces from 77 pigs owned by 16 smallholder pig farmers were randomly collected (manually) par rectum to be examined for intestinal helminths. Sterile plastic gloves were utilised for collection. Sampled pigs were marked to avoid duplicating the samples. The majority of the experimental pigs (*n* = 51) were reared semi-intensively while others were raised intensively (*n* = 26). In the intensive system, the pigs were kept in partially covered stalls and water and feed were placed inside the stalls, while in the semi-intensive system the pigs were allowed to forage for food and water in the open, returning to their stalls at night. Already dewormed pigs (*n* = 24) from both farm management systems were also included to test the efficacy of the anthelmintics used. The sampled pigs were of foreign breeds (Large White or Large White × Landrace). Pigs between 0 and 3 months were noted as piglets (*n* = 25), 3–7 months were growers (*n* = 36) and the pigs above 7 months were recorded as adults (*n* = 16). Moreover, both sexes were taken into consideration, with male pigs accounting for *n* = 32 and female pigs *n* = 45. The number of faecal samples collected depended on the number of pigs available and the number of farms surveyed. The inclusion criteria of the survey farms were accessibility to the farm, the availability of farmers and the voluntary participation of the farmers. Optimum care was taken to avoid additional contamination of the samples. The samples were immediately sent to the laboratory for analysis. Faecal samples were collected between the summer and autumn months of January through March 2016.

### Identification and quantification of helminth eggs

The faecal samples were analysed in the provincial veterinary laboratory of the Department of Agriculture, Bloemfontein. The identification and quantification procedures were based on the standard operating procedure (SOP) of the veterinary laboratory, Bloemfontein, as recommended by Wentzel and Vermeulen (2003). The McMaster counting technique was used to determine the faecal egg count (FEC) per gram of faeces. This technique is used to demonstrate and provide a quantitative estimate of faecal egg output for helminths and coccidia. For the laboratory procedure, the faecal samples were weighed, and two grams of each was placed in an already marked beaker. Fifty-eight millilitres of floatation fluid made up of 40% salt solution was added to the beaker and a glass rod was used to break up the faeces to mix the sample properly. The mixture was left to stand for 5 min and was mixed again with the glass rod. Thereafter, a Pasteur pipette was used to extract aliquots from the faecal mixture to fill the two chambers of the McMaster slide. This was left to settle and stand for about 3 min. Each McMaster slide was examined using a 10 × 10 magnification compound microscope, and the eggs (if present) were identified based on a combination of key structural and morphometric features. Quantification was done by counting all the eggs and oocysts within the engraved areas in the two McMaster chambers, and the total was multiplied by 50. The FEC is usually expressed as eggs per gram (EPG). EPG ≤ 100 was grouped as low levels of infection, EPG > 100 < 500 was regarded as moderate infestation, while EPG ≥ 500 was grouped as significantly high levels. For coccidia oocysts, ≤ 500 oocysts per gram (OPG) was recorded as a low level of infection, while numbers > 500 OPG were regarded as a high level of infection. To further differentiate between similar nematode eggs, coproculture was conducted to obtain the L_3_ stage larva using the Baermann technique (Permin et al. 1999) and the identification was based on the morphological features of the larvae. In cases where trematode eggs were suspected to be present, the sedimentation technique as described in the SOP was done. Differentiation between coccidia species was not done. Appropriate hygiene and safety procedures were properly adhered to.

### Statistical analysis

Data was captured in a Microsoft^®^ Excel version 2016 spreadsheet and was properly coded. Thereafter, it was exported to the IBM SPSS version 22 statistical package for data analysis. Descriptive analyses of percentages, prevalence and pictorial representations were computed using the statistical package and Excel workstations. Relationships between the rate of the parasitic infections of the sampled pigs and risk factors such as farm location, age, sex, farm type and health status were tested using the chi-square test. The level of significance was set at *p* < 0.05. The prevalence of each species of gastrointestinal parasite was calculated as the ratio between the number of infected animals (*n*) and the total number of animals sampled in that category (*N*).

### Ethical considerations

The protocol for this research was evaluated and approved by the Animal Ethics Committee of the University of the Free State, Bloemfontein, South Africa, before the commencement of the survey. The animal experiment number is NR 24/2013.

## Results

In the overall prevalence of gastrointestinal parasites, 61 samples (79.2%) tested positive for one or more parasite species, which were observed as single or mixed infections. Three species of gastrointestinal helminth of veterinary importance and coccidia were identified in the faecal samples (see Table 1).

**TABLE 1:** Overall prevalence of gastrointestinal helminths and parasites in pigs from selected farms in the central Free State Province.

Description	Total number of samples	Number of infected samples	Prevalence of infection (%)
Faecal samples	77	61	79.2
Parasites			
*Ascaris suum*	77	35	44.5
*Trichuris suis*	77	39	50.6
*Oesophagostomum dentatum*	77	2	26.0
Coccidia spp.	77	56	72.7

The highest parasitic load was recorded in Botshabelo (91%), followed by Bloemfontein (83.8%), Thaba Nchu (72.7%) and Mangaung (66.7%), as listed in Table 2. *Ascaris suum* was most prevalent in Mangaung (61.1%) and least prevalent in Thaba Nchu (36.4%), while Botshabelo recorded the highest prevalence for both *T. suis* (72.7%) and *Oesophagostomum dentatum* (36.4%). The prevalence of coccidia was high in all four farming locations. There was a significant difference (*p* < 0.05) in the prevalence of intestinal parasites amongst the farming communities; *T. suis* and coccidia also differed significantly (*p* < 0.05) in prevalence. However, there was no significant difference (*p* > 0.05) in the rate of recovery of *A. suum* and *O. dentatum* eggs from the different farming locations.

**TABLE 2:** Parasitic load enumeration based on farm location, farm practice, age, sex and health status of pigs from selected farms in the central Free State Province.

Variable	Category	*N*	Parasitic load		*Ascaris suum*		*Trichuris suis*		*Oesophagostomum dentatum*		Coccidia spp.	
			*n*	%	*n*	%	*n*	%	*n*	%	*n*	%
FL	Bloem	37	31	83.8	14	37.8	20	54.1	10	27.0	27	73.0
	Botshabelo	11	10	91.0	6	54.5	8	72.7	4	36.4	10	90.9
	T. Nchu	11	8	72.7	4	36.4	4	36.4	0	0	7	63.6
	Manguang	18	12	66.7	11	61.1	7	38.9	6	3.3	12	66.7
	*p*	-	0.001		0.067		0.002		0.247		0.001	
FMP	Intensive	26	19	73.1	3	11.5	4	15.4	1	3.8	13	50.0
	S-intensive	51	42	82.4	32	62.7	35	68.6	19	37.3	43	84.3
	*p*	-	0.003		0.001		0.001		0.001		0.001	
Age	Piglets	25	22	88.0	5	20.0	6	24.0	2	8.0	22	88.0
	Growers	36	28	77.8	23	63.9	23	63.9	11	30.6	27	75.0
	Adults	16	11	68.8	7	43.8	10	62.5	7	43.8	7	43.8
	*p*	-	0.026		0.001		0.002		0.047		0.003	
Sex	Male	32	22	68.8	14	43.8	17	53.1	7	21.9	19	59.4
	Female	45	39	86.7	21	46.7	22	48.9	13	28.9	37	82.2
	*p*	-	0.015		0.237		0.423		0.180		0.016	
HS	Dewormed	24	15	62.5	5	20.8	5	20.8	2	8.3	12	50.0
	ND	53	46	86.8	30	56.6	34	64.2	18	34.0	44	83.0
	*p*	-	0.001		0.001		0.001		0.001		0.001	

*N*, number of pigs sampled; *n*, number of infected pigs; FL, farm location; FMP, farm management practice; HS, health status; Bloem, Bloemfontein; T. Nchu, Thaba Nchu; S-intensive, semi-intensive; ND, not-dewormed.

Piglets, 0–3 months; growers, 3–7 months; adults, 7+ months.

*p* < 0.05.

In Table 2, results by farm type reveal that the intensive and semi-intensive management systems recorded divergent prevalence rates for intestinal parasites. Although the data in this table showed that the percentages were close, a statistically significant difference (*p* < 0.05) was found in the rate of parasitic infection between the two farm types, with 73.1% and 82.4% for the intensive and semi-intensive systems, respectively.

This study detected the highest prevalence of intestinal parasites in piglets (88%), while adult pigs recorded the lowest prevalence (68.8%) (see Table 2). There was a significant difference (*p* < 0.05) in the prevalence of intestinal parasites across all reported parameters in the age category.

The same parasite species were found in males and females, although more study is needed regarding the coccidian species to see if they are all one species or different species (see Table 2). However, female pigs recorded a higher (86.7%) rate than male pigs (68.8%), and a significant difference (*p* < 0.05) was observed in their rates of infection.

Table 2 further illustrates a higher prevalence of intestinal parasites amongst the pigs that had not been dewormed (86.8%) than the dewormed group (62.5%). The overall prevalence of parasites in both groups of pigs and in all noted parameters differed significantly (*p* < 0.05).

Pigs in this study excreted mostly low (EPG ≤ 100) to moderate (EPG > 100 < 500) levels of helminth eggs in all the farm areas (see Table 3). However, few samples in Bloemfontein exhibited high levels (EPG ≥ 500) of *A. suum* and *T. suis* eggs. Coccidia oocytes recorded high levels (OPG > 500) in almost all the farm areas.

**TABLE 3:** The intensity of gastrointestinal parasite infections in pigs from selected farms in the central Free State Province.

Farm area	Intensity of infection (EPG/OPG)			
	*Ascaris suum*	*Trichuris suis*	*Oesophagostomum dentatum*	Coccidia spp.
Bloemfontein	+++	+++	+	+++
Botshabelo	+	++	+	+++
Thaba Nchu	+	++	-	++
Mangaung	++	+	+	+++

Note: For coccidia spp.: ++, moderate infestation (OPG > 100 ≤ 500); +++, high infestation (OPG > 500).

OPG, oocysts per gram; EPG, eggs per gram.

-, No observed infection; +, low infestation (EPG ≤ 100); ++, moderate infestation (EPG > 100 < 500); +++, high infestation (EPG ≥ 500).

**FIGURE 4:**
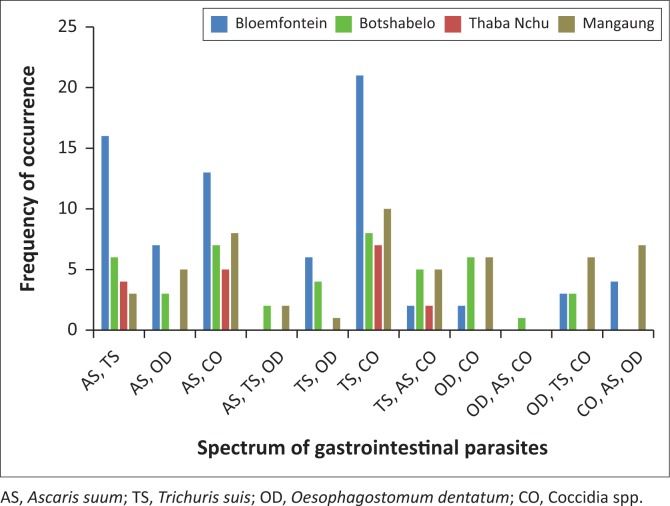
Mixed spectrum of gastrointestinal parasites in faecal samples of pigs from selected farms in the central Free State Province.

The occurrence of mixed parasitic infections recorded 11 different associations of the identified parasites. These associations ranged from double to triple parasitic mixed infections (see Figure 4).

## Discussion

Pig production can be profitable by yielding rapid returns on capital investments; however, internal parasitism is one of the limiting factors that impact a profitable piggery enterprise (Nsoso et al. 2000). In addition, some of these pig helminths are zoonotic in nature and can infect humans (FAO 2008; Spencer 2010). In this study, we investigated the prevalence of gastrointestinal helminth parasites in pigs raised by smallholder farmers in the central Free State. There was a high overall prevalence (79.2%) of intestinal parasites and coccidia species. Similar overall prevalence of intestinal parasites in pigs had been recorded in Nigeria (Sowemimo et al. 2012 [80.4%]), Korea (Ismail et al. 2010 [73.4%]) and Kenya (Obonyo et al. 2012 [83%]). Higher prevalence of 91% (Nissen et al. 2011), 92.7% (Tamboura et al. 2006), 94% (Waiswa et al. 2007) and 96.4% (Dey et al. 2014) was also reported in Kabale District, Uganda; Burkina Faso; south-eastern Uganda; and Bangladesh, respectively. However, in this study the prevalence of intestinal parasites in pigs was higher than the results obtained in Ethiopia (25%) and in Zimbabwe (58.7%) by Jufare et al. (2015) and Marufu et al. (2008), respectively. These recorded results probably varied because of geographical and climatic conditions, various pig breeds, farm management practices, the nutritional and health status of the pigs, method of sample collection and analysis, and differences in the number of samples analysed.

The overall prevalence (44.5%) of *A. suum* in this study was similar to the prevalence recorded for other studies, namely: 40% by Tamboura et al. (2006) in Burkina Faso, 50.9% by Dey et al. (2014) in Bangladesh and 54.6% by Nsoso et al. (2000) in Botswana. However, varied results for *A. suum* had been recorded across a variety of locations, which may be because of seasonal and geographic variations that favour the proliferation of the helminth. To explain this phenomenon, Kagira (2010) and Obonyo et al. (2012) argued that perpetual wet farm conditions, an unhygienic environment and favourable temperatures can lead to high infection rates with *A. suum*. The eggs can withstand adverse weather conditions and some chemicals, and they may remain viable and infective for extended periods (Roepstorff & Nansen 1998). According to Leman et al. (1986), the cause of ‘milk spot’ liver in growing pigs is because of the larval migration of *A. suum*. Polley and Mostert (1980) found that a reduction in weight of up to 40% occurred in pigs infected with *A. suum* and a reduction of up to 25% occurred in feed conversion efficiency.

The whipworm (*T. suis*) was the most prevalent (50.6%) of all the helminths recovered in this study. This result is higher than some previous studies by Jufare et al. (2015), Marufu et al. (2008), Nsoso et al. (2000) and Obonyo et al. (2012), who reported 2.9% in Ethiopia, 4.7% in Zimbabwe, 6.8% in Botswana and 7.8% in Kenya, respectively. However, the result is slightly similar to the 38% and 37.5% prevalence reported respectively in the West Indies (Tiwari et al. 2009) and in outdoor pigs in the Netherlands (Eijck & Borgsteede 2005). The higher prevalence of *T. suis* in this study might have been because of the poor management and husbandry practices that were observed on most of the farms that were visited. Another explanation may be that whipworm eggs are hardy and can therefore withstand adverse environmental conditions for up to 4 years (Urquhart et al. 1996). Heavy infestations are more common in growing and adult pigs raised outdoors, and heavy infestation usually presents as bloody diarrhoea (Roepstorff & Nansen 1998). *Trichuris suis* can be zoonotic (Leman et al. 1986) and is therefore a public health concern.

*Oesophagostomum dentatum* (nodular worm) was recovered at an overall prevalence of 26%. Similar to the result of this study, Eijck and Borgsteede (2005) observed 25%, 27.2% and 22.2% of this worm on free range, organic and conventional farms in the Netherlands, respectively. Moreover, infestation was reported at 37% in Kenya, 27.6% in India and 27.6% also in Kenya by Kagira et al. (2012), Yadav and Tandon (1989) and Kagira et al. (2002), respectively. These results concur with the result obtained in this study. However, divergent results were obtained by Obonyo et al. (2012), Tiwari et al. (2009), Dey et al. (2014), Marufu et al. (2008) and Tamboura et al. (2006), who observed a prevalence of 74% in Kenya, 44% in the West Indies, 12.7% in Bangladesh, 14% in Zimbabwe and 15.6% in Burkina Faso, respectively. These discrepancies may have been partly because of seasonal and geographical variations, pig breed, health status and effective management practices, or the lack thereof.

Coccidia was the most recovered parasite, at 72.7% prevalence. This finding compares well with that of similar investigations in the West Indies where coccidia spp. was the most recovered intestinal parasite (Tiwari et al. 2009). Based on a study in Bangladesh, Dey et al. (2014) reported a combined coccidia spp. prevalence of 65.5% (*Eimeria* spp. and *Isospora suis*); and based on studies on organic and conventional farms in the Netherlands, Eijck and Borgsteede (2005) reported a prevalence of 90.9% and 66.7% of coccidia, respectively. However, the findings of other studies did not concur with the present study. For example, Obonyo et al. (2012), Jufare et al. (2015) and Abdu and Gashaw (2010) recovered coccidian oocytes at 34.8% in Homabay District, Kenya; 12% in Bishoftu, Ethiopia; and 5.6% around Holeta, Ethiopia, respectively. These variations may have been a result of different husbandry management practices in the various study areas, the season of sample collection, pig breed, general health status of the sampled pigs, sample size and so on.

Following the parasitic load observed in the Botshabelo (91%), Bloemfontein (83.8%), Thaba Nchu (72.7%) and Mangaung (66.7%) farm areas, *A. suum* was most prevalent in Mangaung (61.1%) and least prevalent in Thaba Nchu (36.4%). Botshabelo recorded the highest prevalence for both *T. suis* (72.7%) and *O. dentatum* (36.4%). The prevalence of coccidia was high in all four farming locations. Because there were no previously published results that could be traced to compare with helminth infection rates in these geographical areas, it is suggested that sample size, distance to laboratory and prevalent farm practice could be linked to the variations in the rates of gastrointestinal parasitic infections. Moreover, more faecal samples were collected from semi-intensively managed pigs in Botshabelo, and this may account for the higher prevalence of gastrointestinal parasites that was observed there. There is evidence in the literature to suggest that outdoor swine production systems are more susceptible to intestinal parasite problems than indoor systems (Nansen & Roepstorff 1999; Roepstorff & Jorsal 1989).

Although results were statistically significant (*p* < 0.05), the close difference in the prevalence of intestinal parasites in both the intensive (73.1%) and semi-intensive (82.4%) management systems could be linked to the poor sanitary and biosecurity measures that were observed on both farm types. In addition, pigs raised intensively were sometimes allowed to forage outside in times of feed scarcity or to alleviate their hunger during dry seasons. This must have exposed them to almost the same kind of gastrointestinal parasites and intensity of infection as the free rangers. This may account for the similar results in this category compared with reports from Homabay District, Kenya (Obonyo et al. 2012); Busia District, Kenya (Kagira 2010); and in Uganda (Nissen et al. 2011), where respective prevalences of 83%, 84.2% and 91% were obtained for scavenging, free range or extensively raised pigs. The semi-intensive farms had a higher incidence of all four gastrointestinal parasites when compared with the intensive farms. This finding supports previous reports such as the one by Liu and Lu (2002), who stated that the gastrointestinal parasite burden of intensively managed pigs is usually lower.

In the age category, the highest rate of parasitic egg isolation was recorded for piglets at 88%, while adult pigs recorded the lowest (68.8%). In their experiment in Homabay District, Kenya, Obonyo et al. (2012) also noted that the lowest prevalence of helminth infections (79%) occurred in adult pigs, unlike Jufare et al. (2015) in Ethiopia, who reported the lowest prevalence of 19.9% in piglets. Similarly, Bugg et al. (1999) in Western Australia; Tiwari et al. (2009) in Grenada, the West Indies; Lai et al. (2011) in Chongqing, China; and Sowemimo et al. (2012) in Ibadan, Nigeria, reported the highest prevalence in piglets. Other reports disagree with the findings of the current study by observing that the highest prevalence of intestinal helminths occurred amongst growers and/or adult pigs (Dey et al. 2014; Dutta et al. 2005; Nsoso et al. 2000; Roepstorff & Nansen 1998) in Nordic countries, Botswana, India and Bangladesh, respectively. The current study found that age had a significant influence (*p* < 0.05), and it is a risk factor on the prevalence of gastrointestinal parasites.

Recovered parasites were higher in females (86.7%) than in males (68.8%). However, both sexes were infected, with each of the gastrointestinal parasites identified in this study. Recent reports by Dey et al. (2014) and Jufare et al. (2015) confirmed a higher parasitic prevalence in female pigs than in male pigs in Bangladesh and Ethiopia, respectively. Moreover, studies in Burkina Faso (Tamboura et al. 2006), Nigeria (Opara et al. 2006) and Kenya (Obonyo et al. 2012) concurred that females shed significantly more helminth eggs than males, but these studies disagree with Kagira (2010) and Sowemimo et al. (2012), who reported a higher parasitic prevalence in male pigs in Kenya and Nigeria, respectively. Conversely, an earlier report by Yadav and Tandon (1989) observed no significant difference in parasitic infestation between male and female pigs. In females, factors such as hormonal imbalance, gravidity, parturition, lactation and stress, all of which generally alter the physiologic state of female pigs, may lead to supressed immunity and a predisposition to pathogens could be responsible for the higher prevalence (Lloyd 1983; Swai et al. 2010).

There was a higher prevalence of gastrointestinal parasites amongst the pigs that had not been dewormed (86.8%) than the dewormed group (62.5%). Coccidia was the most prevalent parasite in both categories at 50% for dewormed and 83% for non-dewormed pigs. Poor management and unsanitary conditions in the pigpens on most of the farms surveyed could account for the high rate of coccidia infection, especially in already medicated pigs. The issue of over-reliance and indiscriminate use of anthelmintics play an important role in the perpetuation of gastrointestinal helminths as it results in the eventual resistance of these nematodes to the medication. Earlier studies have detected resistance to pyrantel, levamisole and benzimidazoles in *Oesophagostomum* spp. in pigs (Bjørn, Hennessy & Friis 1996; Gerwert, Failing & Bauer 2002; Roepstorff et al. 1987).

There were mostly low (EPG ≤ 100) to moderate (EPG > 100 < 500) levels of helminth eggs observed in all the farm areas. However, few samples in Bloemfontein exhibited high levels (EPG ≥ 500) of *A. suum* and *T. suis* eggs. Coccidia oocytes recovered were high (OPG > 500) in almost all the farm areas. The result of this study correlated with earlier reports from Ghana by Permin et al. (1999) and Burkina Faso by Tamboura et al. (2006), who respectively experienced a high overall prevalence of nematode infections without a corresponding high incidence in EPG. It is possible that the low to moderate intensity of excreted helminth eggs could indicate false negative results because of pre-patent periods of the immature worms or subclinical infections in the sampled pigs. Adebisi (2008) and Marufu et al. (2008) argued that subclinical infections are the most significant form of infection, as they give rise to low productivity of livestock and huge economic losses. Low productivity because of poor feed conversion, piglet mortality, unthriftiness and unprofitable piggery enterprises resulting from very low levels of economic return, were experienced in the study region. Hence, helminthiasis could be a contributing factor associated with these challenges that are faced by smallholder farmers. Weather conditions could also give rise to this pattern of intensity. There had been very little rain before the samples were collected because of the drought and the resultant low levels of precipitation that was experienced in South Africa at the time of the study. The dry environmental conditions may thus not have favoured the development and proliferation of helminth eggs, as posited by Tamboura et al. (2006). The number of samples that were analysed and the season of collection may also have contributed to the low to moderate EPG intensities despite the high prevalence of gastrointestinal parasites.

The double to triple mixed parasitic infections detected was similar to that of an earlier report from Denmark by Roepstorff and Jorsal (1989), and it also corroborated the findings of later studies in Burkina Faso (Tamboura et al. 2006), Nigeria (Sowemimo et al. 2012) and Ethiopia (Jufare et al. 2015). These studies identified multiple (double-quadruple) mixed associations of intestinal parasites, thereby confirming the occurrence of polyparasitism in pigs that are exposed to the outdoors.

## Conclusion

This study showed that different species of gastrointestinal parasites are present in most pigs reared by smallholder farmers in the study area. Outdoor pigs are more susceptible to these parasites than their indoor counterparts. The risk factors for contracting and harbouring some of the identified parasites are age, sex, management system and geographical location. Further in-depth studies that will survey parasitic infections during all four seasons using larger sample populations are required to ascertain the levels of helminth and parasite contamination on smallholder farms in this province. This will lead to employing appropriate solutions to curb the high rate of failed piggery enterprises resulting from unprofitability. Every effort should be made to ensure that smallholder pig farmers get higher returns for their products and that the safety and wholesomeness of the pork they produce are guaranteed for the consumer.
